# Woven Coronary Artery Disease Successfully Managed with Percutaneous Coronary Intervention: A New Case Report

**DOI:** 10.1155/2015/516539

**Published:** 2015-09-30

**Authors:** Yakup Alsancak, Burak Sezenoz, Sedat Turkoglu, Adnan Abacı

**Affiliations:** ^1^Department of Cardiology, Ataturk Education and Research Hospital, Bilkent, 06800 Ankara, Turkey; ^2^Department of Cardiology, Gazi Mustafa Kemal State Hospital, Gazi Mahallesi, 06500 Yenimahalle, Turkey; ^3^Department of Cardiology, Gazi University Medical Faculty, Besevler, 06500 Ankara, Turkey

## Abstract

Woven coronary artery is relatively rare and can be complicated in both acute and chronic phases. A few case reports have been published until now. Herein we report a case with right woven coronary artery managed with drug-eluted stent implantation without complication.

## 1. Introduction

Woven coronary artery (WCA) disease is an extremely rare congenital anomaly with unexplained etiology [1]. In this malformation a part of epicardial coronary artery is divided into many long and thin channels. Thereafter these channels merge again in order to form the main coronary lumen after twisting along anomalous artery axis [2]. Previously published cases about this subject have shown that this anomaly may affect both right and left coronary artery (LAD). Normal blood flow after anomalous coronary segment secures related territory; thus this condition is considered to be benign [3]. Herein, we report a case of WCA in right coronary artery (RCA) successfully managed with percutaneous coronary intervention (PCI) after abnormal myocardial perfusion scintigraphy.

## 2. Case Report

**Figure 1:**
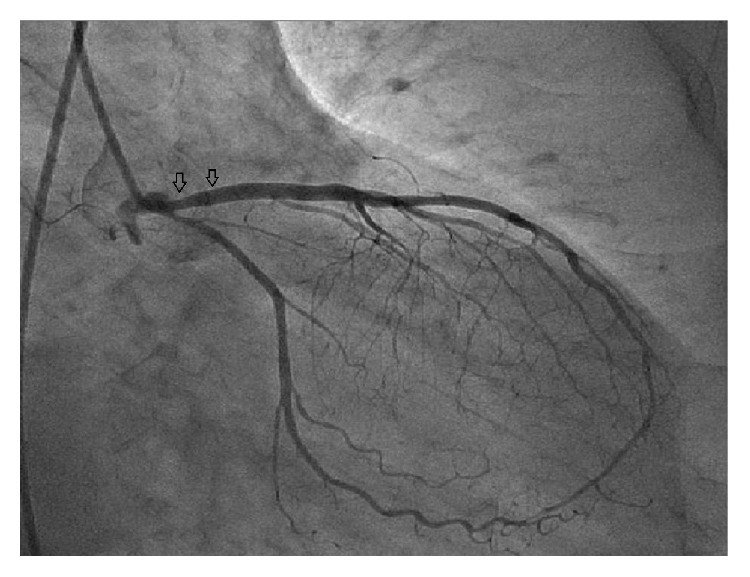
Plaques at left anterior descending artery and borderline lesion ostial Circumflex artery.

**Figure 2:**
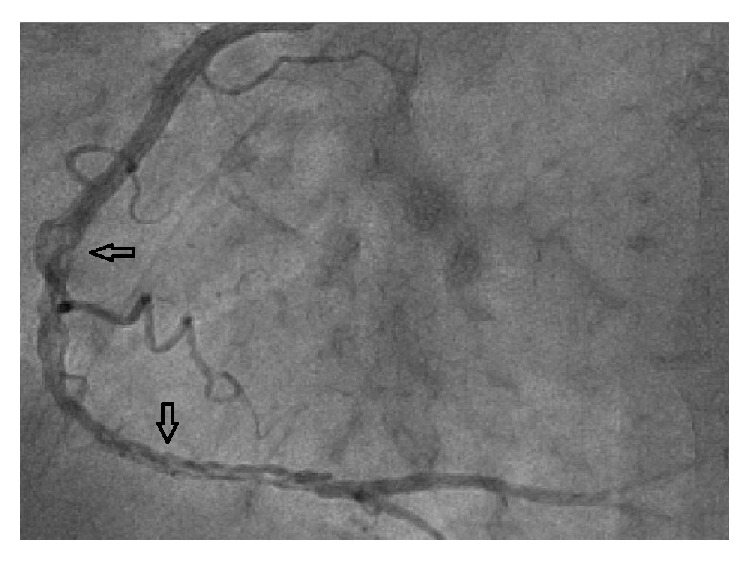
Woven coronary artery anomaly at the midsegment of the right coronary artery.

**Figure 3:**
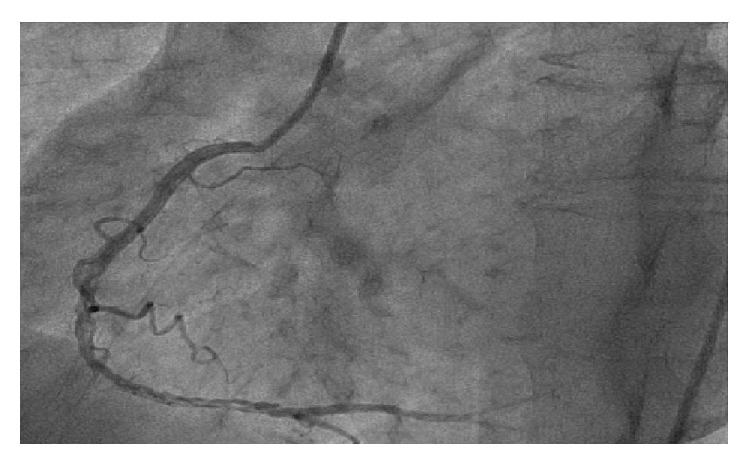
There is normal blood flow at the distal RCA segment of the anomaly.

**Figure 4:**
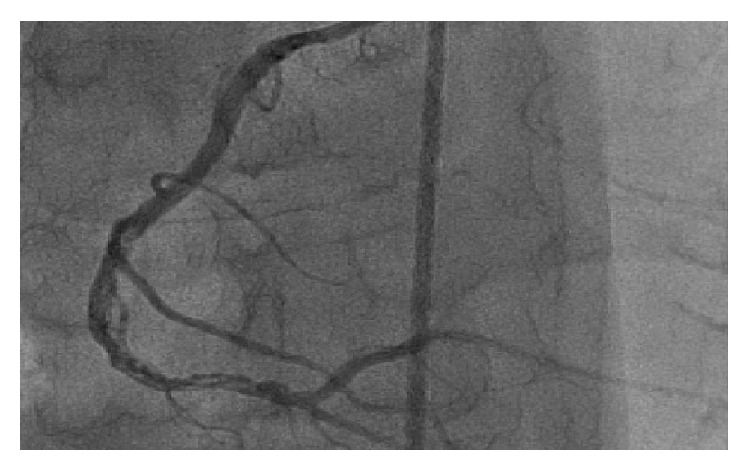
Coronary angiography showed proximal thin channels and distal reanastomosis.

**Figure 5:**
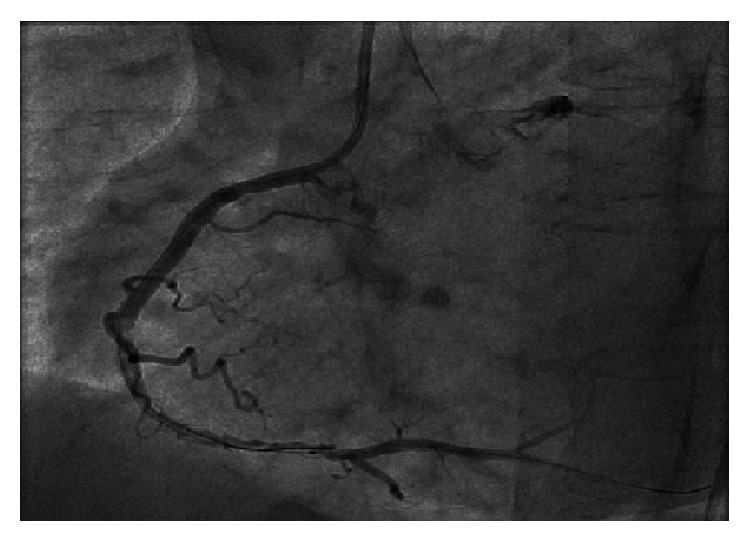
Right coronary artery and TIMI III flow after floppy guidewires.

**Figure 6:**
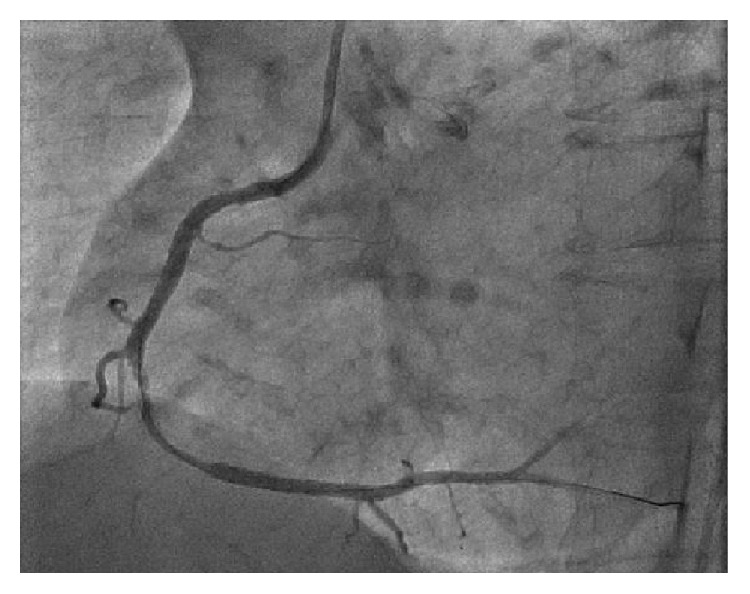
Right coronary artery and WCA after first distal stent implantation.

**Figure 7:**
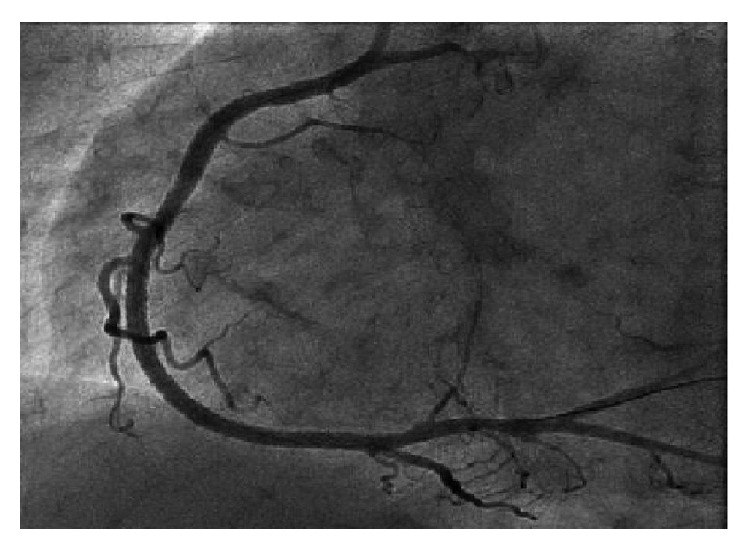
RCA after percutaneous coronary intervention and distal normal blood flow.

54-year-old male with tightening chest pain and palpitation on exertion for two months was admitted to cardiology outpatient clinic two years ago. Resting electrocardiogram showed q waves and extra systoles on D3 and aVF. Echocardiography showed akinesia at inferior and posterior walls, and ejection fraction was 44%. We performed coronary angiography by using the Judkins technique from right femoral artery. There were plaques at LAD and %50 stenosis at proximal Circumflex arteries, the lesions were considered to be insignificant (Figure 1), and the patient had woven RCA (Figures 2 and 3). At nearly 5 cm middle portion of RCA, numerous small tortious channels formed the lumen and they were merged again after the anomalous segment (Figure 4). There was TIMI III flow at the distal part of the anomalous segment. No other lesion was found to explain the patient's complaints; therefore thallium-myocardial perfusion scintigraphy was performed in order to assess ischemic burden of RCA territory. Inferior wall ischemia was detected which approximately refers to %14 of left ventricle. Optimal anti-ischemic treatment did not relieve anginal symptoms and nonsustained ventricular tachycardia episodes were detected in Holter ECG recording that led us to PCI to anomalous RCA portion. Woven pattern was confirmed at RCA with no apparent coronary stenosis. 6F guiding catheter was placed to RCA ostium and then 0.014 floppy guiding wire was forwarded to anomalous portion (Figure 5). 1.5/15 mm chronic total occlusion angioplasty balloon was used to reach lesion. Control imaging showed protected TIMI III flow in RCA. After dilatation, two 2.5/28 mm everolimus-eluted stents were implanted to the anomalous portion after balloon dilatation (Figures 6 and 7). TIMI III flow was ensured after stent implantation.

## 3. Discussion

Woven coronary artery (WCA) is a very rare congenital anomaly which can affect both RCA and LAD and may lead to acute coronary syndromes in some circumstances [4]. It was first described twenty-five years ago and only a few cases have been reported [2]. There is a male predominancy and only one case of a child has been reported. The distance of abnormal part is limited a few centimeters and blood flow is similar before to the diseased area [3]. Yldrm and his colleagues were speculating that this pathology may be caused by recanalisation of a thrombus [4]. The differential diagnosis should include recanalized thrombus, spontaneous coronary artery dissection, and bridging collaterals [3, 4].

This anomaly may be accepted as a benign disease. Some reports showed no cardiovascular adverse events during 5-year follow-up [3–5]. Nevertheless some authors stated that this anomaly can be associated with myocardial ischemia and damage in their cases [6, 7]. In our case, the optimal medical treatment was started after the first coronary angiography but the chest pain persisted. Because of refractory angina and inferior wall ischemia in myocardial perfusion imaging, PCI was performed to the WCA segment of right coronary artery. Before this report, an inferior myocardial infarction due to WCA disease was reported, but PCI was not performed because the coronary artery was affected extensively [7]. Intravascular ultrasound (IVUS) can be a useful technique in order to manage stent position and to verify adequate stent expansion in these types of patients. Moreover in a patient with multiple vessel disease who underwent bypass surgery and the other who underwent aortic valve operation with woven anomaly has not been revascularized [3, 8]. To our knowledge, only one case has been reported about successful PCI for WCA [9].

## 4. Conclusion

In conclusion, WCA may cause myocardial ischemia and damage. All interventional cardiologists might keep this anomaly in mind and should beware of the possible consequences of WCA in catheterisation laboratory. PCI or coronary artery bypass grafting may be treatment options for this anomaly.
